# Sphingomyelin synthase 1 as a potential upstream amplifier of microglial CSF1R signaling in neuropathic pain

**DOI:** 10.3389/fnmol.2026.1798921

**Published:** 2026-04-20

**Authors:** Minghui Tan, Meiping Wu, Meikui Wu

**Affiliations:** 1 Department of Encephalopathy, Chongqing Jiulongpo Hospital of Traditional Chinese Medicine, Chongqing University of Chinese Medicine, Chongqing, China; 2 Department of Orthopedics, Chongqing Jiulongpo Hospital of Traditional Chinese Medicine, Chongqing University of Chinese Medicine, Chongqing, China; 3 Department of Anesthesiology, National Cancer Center/National Clinical Research Center for Cancer/Cancer Hospital, Chinese Academy of Medical Sciences and Peking Union Medical College, Beijing, China

**Keywords:** CSF1R, diacylglycerol, microglia, neuropathic pain, protein kinase D, sphingomyelin synthase 1

## Abstract

Neuropathic pain is a debilitating chronic condition sustained by maladaptive neuroimmune interactions within the central nervous system, with microglial activation in the spinal dorsal horn serving as a critical driver of pain initiation and chronification. Within this framework, microglial activation mediated by colony-stimulating factor 1 receptor (CSF1R) signaling has emerged as a pathway that is both necessary and sufficient for the development and maintenance of neuropathic pain; however, the upstream mechanisms that determine CSF1R membrane availability and signaling intensity in microglia remain poorly defined. Here, we propose the hypothesis that sphingomyelin synthase 1 (SMS1) functions as a metabolic gatekeeper that amplifies microglial CSF1R signaling by regulating diacylglycerol (DAG)/protein kinase D (PKD)-dependent receptor trafficking. Following peripheral nerve injury, SMS1 expression is upregulated in spinal microglia, leading to increased Golgi-associated DAG production and subsequent PKD activation. Activated PKD promotes vesicular transport of CSF1R from the Golgi apparatus to the plasma membrane, thereby increasing CSF1R surface density and prolonging receptor signaling. Enhanced CSF1R membrane availability amplifies CSF1-driven microglial proliferation and neuroinflammatory signaling, ultimately facilitating synaptic dysregulation and persistent pain hypersensitivity. This hypothesis establishes a direct mechanistic link between sphingolipid metabolism and microglial neuroimmune signaling and identifies SMS1 as a previously unrecognized upstream regulator of neuropathic pain. Targeting SMS1 may therefore represent a novel therapeutic strategy that modulates microglial activation while avoiding the systemic immune suppression associated with direct CSF1R blockade. If validated, this lipid-regulated trafficking mechanism may have broader implications for other microglia-dependent disorders of the central nervous system.

## Introduction

Neuropathic pain represents a major clinical challenge, affecting approximately 7–10% of the general population (Colloca et al., 2017). Characterized by mechanical allodynia, thermal hyperalgesia, and spontaneous pain, it arises from a lesion or disease of the somatosensory nervous system (Rosner et al., 2024). Current pharmacotherapies, including gabapentinoids, tricyclic antidepressants, and SNRIs, offer partial relief to fewer than 50% of patients and are often limited by systemic side effects (Sokol et al., 2025; Soliman et al., 2025). This therapeutic ceiling stems from our incomplete understanding of the molecular mechanisms that sustain the “chronification” of pain signals in the spinal dorsal horn.

Over the past two decades, the focus of pain research has expanded beyond the neuron to include non-neuronal cells (Inoue and Tsuda, 2018; Tsuda et al., 2023). Spinal microglia, the resident immune cells of the CNS, are now recognized as indispensable drivers of central sensitization (Inoue and Tsuda, 2018; Tsuda et al., 2023). Following peripheral nerve injury (PNI), dorsal horn microglia undergo a rapid phenotypic transformation—termed “activation”—marked by proliferation, morphological changes, and the release of pro-algesic mediators such as brain-derived neurotrophic factor (BDNF) and tumor necrosis factor-alpha (TNF-α) (Boakye et al., 2019; Inoue and Tsuda, 2018; Tsuda et al., 2023).

Among the myriad signals regulating microglia, the colony-stimulating factor 1 (CSF1) axis is paramount (Guan et al., 2016; Okubo et al., 2016). Guan et al. (2016) definitively demonstrated that following nerve injury, CSF1 is transported anterogradely from injured sensory neurons to the spinal cord. Here, it binds to CSF1R, a receptor tyrosine kinase expressed almost exclusively by microglia in the CNS (Guan et al., 2016). CSF1R activation is a critical prerequisite for nerve-injury–induced microglial proliferation and the development of mechanical hypersensitivity: genetic deletion of CSF1 in neurons or pharmacological blockade of CSF1R completely abolishes nerve-injury-induced microglial proliferation and the development of mechanical hypersensitivity (Guan et al., 2016).

Given the central role of CSF1–CSF1R signaling in microglial activation (Guan et al., 2016; Okubo et al., 2016), the cellular mechanisms regulating CSF1R availability are likely to be critical determinants of signal strength and biological outcome. For CSF1R to function as an effective signaling receptor, it must be properly localized to the plasma membrane, where ligand binding and receptor activation occur (Miaczynska, 2013). The level of CSF1R at the cell surface is controlled not only by transcriptional and translational processes, but also by post-translational modification and intracellular trafficking of the mature receptor from the Golgi apparatus to the plasma membrane (Bergeron et al., 2016; Cypher et al., 2016; Miaczynska, 2013). However, the molecular mechanisms governing the transport of mature CSF1R from the Golgi apparatus to the plasma membrane remain poorly understood.

SMS1, localized to the trans-Golgi, generates DAG, which serves as a key signaling lipid required for PKD activation and efficient cargo export (Subathra et al., 2011; Villani et al., 2008). Increasing evidence indicates that the sphingomyelin synthase 1 (SMS1)–diacylglycerol (DAG)–protein kinase D (PKD) pathway plays a critical role in regulating protein trafficking from the trans-Golgi network to the plasma membrane. Previous studies have demonstrated that SMS1-dependent DAG/PKD signaling governs the secretion and surface delivery of diverse proteins, including viral glycoproteins, reporter proteins, and membrane ion channels (Subathra et al., 2011; Tafesse et al., 2013). Consistent with this, our prior work showed that SMS1 regulates the plasma membrane expression of the KCNQ1/KCNE1 channel complex via this pathway (Wu et al., 2016). However, whether SMS1-mediated Golgi trafficking contributes to the regulation of CSF1R surface expression in microglia has not yet been explored.

## Hypothesis

**Figure 1:**
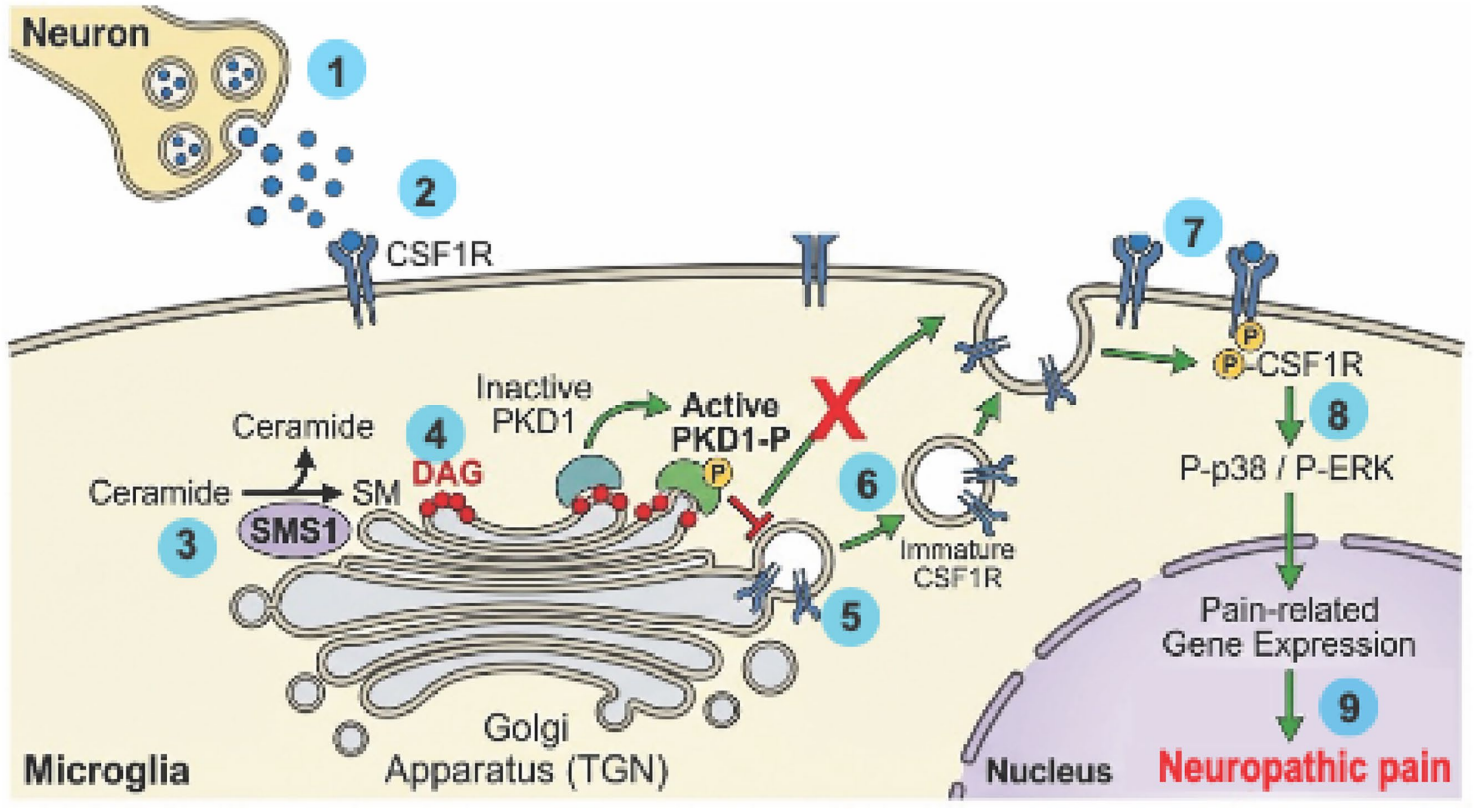
Proposed mechanism of SMS1–PKD1-mediated CSF1R trafficking in neuropathic pain. Following peripheral nerve injury ①, neuron-derived CSF1 triggers a “primed” state in spinal microglia ②. We hypothesize that upregulated SMS1 activity at the trans-Golgi network (TGN) ③ leads to increased local DAG levels, which recruits and activates PKD1 ④. Active PKD1 facilitates the budding and scission of CSF1R-containing vesicles from the TGN ⑤, driving their prioritized forward delivery to the plasma membrane ⑥. This resultant increase in receptor surface density ⑦ amplifies downstream p38/ERK signaling ⑧, ultimately contributing to the persistence of neuropathic pain ⑨. The red cross indicates that pharmacological or genetic inhibition of the SMS1–PKD1 axis disrupts vesicle scission and the subsequent surface delivery of CSF1R, thereby attenuating microglial hypersensitivity.

We hypothesize that activation of the SMS1–DAG–PKD1 signaling axis facilitates the prioritized forward trafficking of newly synthesized CSF1R to the plasma membrane, thereby driving microglial hypersensitivity and the persistence of neuropathic pain (Figure 1). Central to the hypothesis is the role of SMS1 activity in generating a localized, high-concentration pool of diacylglycerol (DAG) at the trans-Golgi network (TGN) membrane, which is required for efficient vesicle biogenesis and membrane remodeling at the trans-Golgi network (Subathra et al., 2011; Tafesse et al., 2013; Wu et al., 2016). Notably, this hypothesis extends prevailing models of microglial sensitization by proposing that post-translational trafficking efficiency represents an additional, and potentially limiting, regulatory layer beyond transcriptional control (Depp et al., 2025; Vecchiarelli and Tremblay, 2023). Instead, it postulates that post-translational trafficking efficiency—rather than transcriptional output—is the true determinant of cellular sensitivity (Cypher et al., 2016; Miaczynska, 2013). In this model, CSF1R is uniquely susceptible to this pathway because the rapid proliferation of microglia creates an exceptionally high demand for new surface receptors (Barry-Carroll and Gomez-Nicola, 2024), making the SMS1-PKD axis the “bottleneck” or rate-limiting step for the pain phenotype.

Mechanistically, the hypothesis suggests that peripheral nerve injury triggers an upregulation of SMS1, which catalyzes the transfer of phosphocholine to ceramide at the TGN. This metabolic signaling generates a DAG pool that acts as a beacon, binding to the C1 domains of cytosolic protein kinase D (PKD) and recruiting it to the organelle (Baron and Malhotra, 2002). Upon activation, PKD phosphorylates key downstream effectors, including PI4KIIIβ and the lipid transfer protein CERT (Baron and Malhotra, 2002; Malhotra and Campelo, 2011), which are required for efficient vesicle biogenesis and membrane remodeling at the trans-Golgi network. Activation of this pathway is expected to facilitate the forward trafficking of newly synthesized CSF1R to the plasma membrane, thereby increasing receptor surface density and enhancing microglial responsiveness to neuronal CSF1 (Cypher et al., 2016). Sustained amplification of CSF1R signaling may, in turn, promote prolonged activation of downstream p38/ERK pathways and contribute to the persistence of neuropathic pain (Guan et al., 2016; Inoue and Tsuda, 2018).

### Evolution of the hypothesis

The biological plausibility of this hypothesis relies on integrating foundational findings from lipid biochemistry and neuroimmunology. This hypothesis is unprecedented in its proposal that a specific sphingolipid metabolic enzyme acts as the “bottleneck” for microglial receptor presentation during chronic pain. While current literature acknowledges the role of microglial activation in neuropathic pain (Boakye et al., 2019; Guan et al., 2016; Inoue and Tsuda, 2018; Tsuda et al., 2023), the mechanistic link between Golgi-specific lipid signaling and receptor trafficking efficiency remains largely unexplored.

Central to the evolution of this hypothesis is the evidence regarding cellular diacylglycerol (DAG) compartmentalization. It is well-established that cellular DAG pools are spatially organized (Ueda et al., 2015); DAG generated at the plasma membrane by PLC signals distinctly from DAG generated at the Golgi (Eichmann and Lass, 2015; Ueda et al., 2015). Notably, studies have demonstrated that SMS1 is the primary source of Golgi-associated DAG, and that pharmacological or genetic blockade of SMS1 creates a “traffic jam” effect where proteins accumulate within the Golgi apparatus instead of progressing to the cell surface (Tafesse et al., 2013; Villani et al., 2008).

The role of protein kinase D (PKD) as the “fission engine” further supports this model. Baron and Malhotra (2002) demonstrated that PKD recruitment to the trans-Golgi network (TGN) is strictly dependent on the localized DAG pool. Furthermore, research by Malhotra and Campelo (2011) elucidated that PKD activity is essential for the scission of transport carriers destined for the cell surface, providing a mechanical basis for our proposed trafficking axis.

Our hypothesis reinterprets existing metabolomic data in the context of neuropathic pain. Previous studies in rodent models have identified significant perturbations in spinal sphingomyelin and ceramide levels following nerve injury (Patti et al., 2012; Wang et al., 2024). While these changes were originally interpreted merely as markers of cellular stress or apoptosis, the present hypothesis postulates that these lipid fluctuations are active drivers of receptor trafficking. Specifically, as a glycosylated receptor tyrosine kinase, CSF1R must traverse the Golgi for maturation, and its surface levels are known to be highly dynamic and regulated by exocytic rates in myeloid cells (Bergeron et al., 2016; Cypher et al., 2016; Miaczynska, 2013).

A potential concern is whether SMS1-PKD signaling broadly regulates surface protein trafficking, and if so, what justifies a particular focus on CSF1R. We propose that CSF1R, as a receptor tyrosine kinase (RTK) with high biosynthetic demand during microglial proliferation, is disproportionately sensitive to perturbations in Golgi-to-plasma membrane trafficking efficiency. Unlike receptors that rely on robust recycling pathways—such as TREM2, which depends on Vps35/retromer-mediated endosomal recycling (Yin et al., 2016)—or those with relatively stable membrane residence like the GPCR CX3CR1, CSF1R requires continuous *de novo* delivery from the Golgi to compensate for rapid ligand-induced internalization and degradation (Huynh et al., 2012; Lou et al., 2014). In the “primed” state following nerve injury, the burst in microglial proliferation creates an exceptional biosynthetic load, rendering CSF1R uniquely vulnerable to perturbations in Golgi-specific lipid signaling.

Regarding isoform specificity, we focus on SMS1 rather than SMS2 because of their distinct subcellular localizations. SMS1 is localized exclusively to the trans-Golgi network, where it generates the DAG pool required for PKD recruitment and vesicle biogenesis (Huitema et al., 2004; Yeang et al., 2011). SMS2, in contrast, localizes primarily to the plasma membrane due to palmitoylation-dependent targeting (Tani and Kuge, 2009). Subathra et al. (2011) demonstrated that knockdown of SMS1, but not SMS2, impairs protein secretion and surface delivery, confirming that the trafficking function is SMS1-specific.

Rather than considering SMS1 solely as a lipid metabolic enzyme (Subathra et al., 2011; Villani et al., 2008), it may be more appropriately viewed as a rate-limiting regulator that couples the intracellular trafficking capacity of microglia with their responsiveness to extracellular CSF1 signals. Notably, evidence from Brekk et al. (2020) demonstrates that neuroinflammatory stimuli can induce Sgms1 expression in microglia, providing a plausible mechanistic basis for the potential upregulation of SMS1 in the spinal cord following peripheral nerve injury.

### Hypothesis testing

To translate the proposed mechanism from a conceptual model into experimentally supported evidence, a multi-level validation strategy is required, encompassing subcellular signaling organization, receptor trafficking dynamics, and *in vivo* behavioral outcomes.

#### Validation of SMS1 induction following nerve injury

A foundational requirement for this hypothesis is to determine whether Sgms1 expression or SMS1 enzymatic activity is altered in spinal microglia following peripheral nerve injury. While global sphingolipid metabolic perturbations have been documented in neuropathic pain models (Patti et al., 2012; Wang et al., 2024), and neuroinflammation-driven Sgms1 induction has been observed in microglia in other disease contexts (Brekk et al., 2020), direct evidence in the spinal dorsal horn following nerve injury remains lacking.

Publicly available single-cell RNA sequencing datasets (Tansley et al., 2022; Fiore et al., 2022) provide a valuable resource for preliminary assessment of *Sgms1* expression in spinal microglia. However, given the moderate expression level of *Sgms1* and the potential for subpopulation-specific regulation, high-sensitivity spatially-resolved approaches may be required for definitive validation. Future studies should employ RNAscope *in situ* hybridization combined with Iba1 immunohistochemistry, or microglial-specific RiboTag sequencing, to determine whether *Sgms1* is upregulated in spinal microglia following nerve injury. If transcriptional upregulation is not observed, alternative mechanisms—including increased enzymatic activity, altered substrate availability, or changes in protein stability—should be investigated using functional assays such as fluorescent ceramide analog conversion.

#### Establishment of the SMS1–DAG–PKD signaling hierarchy in microglia (*in vitro*)

Prior to *in vivo* investigation, the intracellular signaling hierarchy linking SMS1 activity to PKD recruitment must be established in microglial cells. Because conventional lipid extraction methods disrupt spatial organization, the diacylglycerol (DAG) pool at the trans-Golgi network (TGN) will be visualized using genetically encoded DAG biosensors (Subathra et al., 2011). Microglial cells will be treated with pharmacological SMS1 inhibitors (e.g., D609) or subjected to *Sgms1* knockdown via shRNA (Wu et al., 2016). Disruption of SMS1 activity is expected to reduce localized DAG production at the TGN, thereby impairing PKD recruitment and its subsequent activation. We will assess the loss of this signaling hierarchy through immunofluorescent detection of p-PKD (Ser916) (Grimaldi et al., 2022). Supplementation with a membrane-permeable DAG analog (1,2-dioctanoyl-sn-glycerol) will be used to determine whether PKD recruitment and downstream trafficking defects can be restored (Baron and Malhotra, 2002). A critical experimental consideration is distinguishing the trafficking effects of SMS1-generated DAG from potential contributions of sphingomyelin (SM) synthesis *per se*. To address this, rescue experiments using exogenous DAG analogs (e.g., 1,2-dioctanoyl-sn-glycerol) will be performed following SMS1 inhibition. If DAG is the functionally relevant product, exogenous DAG supplementation should restore PKD recruitment and CSF1R surface delivery even when SM synthesis remains suppressed. Conversely, if SM itself is required, DAG rescue would be incomplete. Additionally, comparison with SMS2 knockdown—which generates SM at the plasma membrane without contributing to Golgi DAG pools (Tani and Kuge, 2009)—would further isolate the DAG-specific trafficking function. If SMS1 knockdown impairs CSF1R trafficking while SMS2 knockdown does not, this would support the hypothesis that Golgi-localized DAG, rather than SM synthesis per se, is the critical variable.

#### Quantification of CSF1R surface delivery and trafficking efficiency

A central requirement of the hypothesis is to distinguish changes in total CSF1R expression from alterations in its surface delivery. To achieve this, primary microglia will be analyzed using three complementary approaches. First, cells will be incubated with membrane-impermeable biotin to selectively label surface-exposed proteins for biochemical quantification (Cypher et al., 2016). Second, flow cytometry (FCM) on non-permeabilized microglia stained with fluorophore-conjugated anti-CSF1R antibodies will enable single-cell quantification of surface receptor levels, assessment of population heterogeneity, and simultaneous verification of microglial identity markers (CD11b, CD45). Finally, high-resolution confocal microscopy will verify intracellular accumulation of CSF1R and its colocalization with the TGN marker TGN38 (Baron and Malhotra, 2002).

#### *In vivo* validation of causality in neuropathic pain mouse models

Validation of this hypothesis *in vivo* would require established mouse models of chronic neuropathic pain, such as the spared nerve injury (SNI) model (Decosterd and Woolf, 2000; Richner et al., 2011) or spinal nerve ligation (SNL) model (Guan et al., 2016; Okubo et al., 2016). To determine whether Sgms1 expression is altered in spinal microglia following nerve injury, RNAscope *in situ* hybridization combined with Iba1 immunohistochemistry would enable cell-type-specific transcript detection (Wang et al., 2012). To establish causality, microglia-selective gene silencing could be achieved through intrathecal delivery of viral vectors encoding shRNA targeting *Sgms1* or *Prkd1*, driven by microglia-selective promoters such as *Tmem119* or *Hexb* (Shah et al., 2022; Aoki et al., 2025). Behavioral outcomes would be assessed using von Frey filament testing for mechanical sensitivity (Guan et al., 2016). If the hypothesis is correct, microglia from shRNA-treated mice would be expected to exhibit reduced surface CSF1R levels, linking behavioral outcomes to receptor availability at the microglial membrane.

Importantly, given the known sex differences in microglial involvement in neuropathic pain (Mapplebeck et al., 2016; Sorge et al., 2015), future validation studies must include both male and female mice. Recent evidence demonstrates that microglial mechanisms predominate in males, whereas T-cell-dependent pathways may be more prominent in females (Fan et al., 2025; Tansley et al., 2022). Whether the SMS1–PKD–CSF1R axis exhibits sex-specific regulation remains an open question that warrants systematic investigation to ensure the translational relevance of the proposed framework.

## Discussion

If experimentally validated, this hypothesis suggests an alternative strategy for modulating neuroinflammation, shifting from broad elimination of immune cells toward selective regulation of their functional responsiveness.

### Modulating microglial sensitivity rather than cell ablation

Current therapeutic approaches targeting the CSF1R pathway predominantly rely on tyrosine kinase inhibitors (TKIs), such as PLX3397 (Okojie et al., 2023; Peng et al., 2025). Although effective, these agents often result in near-complete depletion of the microglial population (Okojie et al., 2023; Peng et al., 2025). Such indiscriminate ablation remains clinically controversial, as microglia play essential roles in debris clearance and synaptic maintenance (Colonna and Butovsky, 2017; Hickman et al., 2018). Regarding the specific question of how SMS1 or PKD blockade would affect CSF1R membrane localization, we predict that inhibition of this pathway would selectively reduce the rate of *de novo* CSF1R delivery to the plasma membrane. This would lead to decreased surface receptor density without affecting receptor internalization or degradation kinetics. Importantly, this mechanism differs fundamentally from PLX-mediated CSF1R kinase inhibition. While PLX compounds block CSF1R signaling directly—eliminating the survival signal required for microglial viability and resulting in massive cell death (Elmore et al., 2014; Okojie et al., 2023)—SMS1 inhibition would reduce receptor availability rather than receptor function. Under this “throttling” mechanism, microglia would retain their existing surface CSF1R and remain viable, but would be less responsive to subsequent CSF1 stimulation. Such an approach aims to attenuate hyperactivation while preserving basal homeostatic functions (Colonna and Butovsky, 2017; Hickman et al., 2018).

### State-dependent specificity of SMS1 inhibition

A central concern in targeting lipid metabolic enzymes is the potential for widespread effects. However, under basal conditions, SMS1 activity supports membrane composition. Following peripheral nerve injury (Giussani et al., 2025; Li et al., 2026), microglia enter a primed state with a markedly increased demand for secretory trafficking, including surface receptors such as CSF1R (Cypher et al., 2016; Guan et al., 2016). This heightened burden renders pathological microglia disproportionately dependent on the DAG–PKD axis. Accordingly, inhibition of SMS1 is predicted to preferentially affect activated microglia, a highly desirable property for therapeutic strategies targeting chronic neuroinflammation (Paolicelli et al., 2022).

### A Golgi-centered regulatory node in immunometabolism

This framework highlights the Golgi apparatus as an active regulatory node. The lipid composition of the TGN emerges as a determinant of whether immune activation signals are efficiently propagated. Although this study focuses on the CSF1–CSF1R loop in neuropathic pain (Guan et al., 2016; Okubo et al., 2016), the concept of a Golgi-based checkpoint may have broader relevance to other inflammatory and neurodegenerative conditions.

## Concluding remarks

In conclusion, we propose that enhanced efficiency of intracellular receptor trafficking in microglia represents an underappreciated contributor to chronic pain. By generating a localized diacylglycerol pool at the trans-Golgi network, SMS1 regulates PKD-dependent transport of CSF1R to the plasma membrane, thereby shaping microglial sensitivity to neuronal injury signals. Elucidation of the SMS1–DAG–PKD–CSF1R axis addresses a fundamental question regarding receptor surface availability and provides a mechanistic foundation for the development of non-opioid therapies targeting the intracellular origins of neuroinflammation.

## Data Availability

The original contributions presented in the study are included in the article/supplementary material, further inquiries can be directed to the corresponding author.
